# Polymer Composites Based on Polycarbonate (PC) Applied to Additive Manufacturing Using Melted and Extruded Manufacturing (MEM) Technology

**DOI:** 10.3390/polym13152455

**Published:** 2021-07-26

**Authors:** Katarzyna Bulanda, Mariusz Oleksy, Rafał Oliwa, Grzegorz Budzik, Łukasz Przeszłowski, Jacek Fal, Teofil Jesionowski

**Affiliations:** 1 Department of Polymer Composites, Faculty of Chemistry, Rzeszów University of Technology, Al. Powstańców Warszawy 6, 35-959 Rzeszów, Poland; molek@prz.edu.pl (M.O.); oliwa@prz.edu.pl (R.O.); 2 Department of Machine Design, Faculty of Mechanical Engineering and Aeronautics, Rzeszów University of Technology, Al. Powstańców Warszawy 12, 35-959 Rzeszów, Poland; gbudzik@prz.edu.pl (G.B.); lprzeszl@prz.edu.pl (Ł.P.); 3 Department of Physics and Medical Engineering, Faculty of Mathematics and Applied Physics, Rzeszów University of Technology, 35-959 Rzeszów, Poland; jacekfal@prz.edu.pl; 4 Faculty of Chemical Technology, Institute of Chemical Technology and Engineering, Poznan University of Technology, 60-965 Poznań, Poland; teofil.jesionowski@put.poznan.pl

**Keywords:** MEM, 3D printing, additive manufacturing, thermoplastic polymer, polycarbonate

## Abstract

As part of the present work, polymer composites used in 3D printing technology, especially in Melted and Extruded Manufacturing (MEM) technology, were obtained. The influence of modified fillers such as alumina modified silica, quaternary ammonium bentonite, lignin/silicon dioxide hybrid filler and unmodified multiwalled carbon nanotubes on the properties of polycarbonate (PC) composites was investigated. In the first part of the work, the polymer and its composites containing 0.5–3 wt.% filler were used to obtain a filament using the proprietary technological line. The moldings for testing functional properties were obtained with the use of 3D printing and injection molding techniques. In the next part of the work, the rheological properties—mass flow rate (MFR) and mechanical properties—Rockwell hardness, Charpy impact strength and static tensile strength with Young’s modulus were examined. The structure of the obtained composites was also described and determined using scanning electron microscopy (SEM). The porosity, roughness and dimensional stability of samples obtained by 3D printing were also determined. On the other hand, the physicochemical properties were presented on the basis of the research results using thermogravimetric analysis (TGA), differential scanning calorimetry (DSC), wide angle X-ray scattering analysis (WAXS) and Fourier Transform infrared spectroscopy (FT-IR). Additionally, the electrical conductivity of the obtained composites was investigated. On the basis of the obtained results, it was found that both the amount and the type of filler significantly affected the functional properties of the composites tested in the study.

## 1. Introduction

Nowadays, additive manufacturing (AM), also known as three-dimensional manufacturing or 3D printing, is an advanced, rapidly evolving manufacturing method that produces physical models, including complex geometric structures, with high accuracy while maintaining low process costs [1,2]. Since its rapid development in the 1990s, 3D printing technology has been widely used to replace the parts that are difficult and expensive to produce with traditional methods, including automotive and aviation applications [3]. Additive manufacturing is a rapidly deployed technology in both domestic and industrial settings due to its many advantages such as reduced design and production cycle time, waste reduction, energy efficiency and the ability to use affordable tools [4,5]. Three-dimensional printing also offers automation and repeatability at a high level [5]. The technology refers to a computer-aided method of manufacturing an element based on the provided three-dimensional design pattern, i.e., using a data file saved in the STL format obtained from the geometric guidelines of objects also with complex geometry [2].

Based on the printing technology, AM can be divided into several basic processing methods, namely material extrusion, powder bed fusion, sheet lamination, binder jetting, direct energy deposition, material jetting and vat photo-polymerization [1,6]. Among the presented methods, the Melted and Extruded Manufacturing (MEM) technology is an extrusion method in which structures and models are obtained from filaments composed of thermoplastic polymers, including acrylonitrile-butadiene-styrene (ABS), polylactide (PLA), poly(ethylene terephthalate) (PET), polyamides (PA) and polycarbonates (PC) [7]. In technology, an object is built by selectively applying a molten material layer by layer along a predetermined path [5]. MEM is characterized by high versatility, relative simplicity and low cost of both tools and input materials [8]. Typically, Melted and Extruded Manufacturing products are used as conceptual prototypes rather than as functional elements, primarily due to the lack of excellent performance properties. The resulting thermoplastic parts have poorer mechanical properties compared to traditional manufacturing methods such as injection molding [7]. These limitations prompted researchers to develop various types of solutions that will allow obtaining MEM parts with increased strength [7,9]. Therefore, scientists conducted a complete study to optimize the main parameters of the process [8], including layer thickness, nozzle temperature, platform temperature, printing speed, raster angle, feed speed and printing orientation. Unfortunately, the correction of printing parameters did not bring the expected results of a significant change in the properties of the part, so now scientists have focused on the appropriate selection of the material and on ways to modify the properties of the materials used [3,10,11]. In particular, polymer matrix composite materials have recently revolutionized the materials industry thanks to their outstanding properties [11].

One of the many thermoplastics used in 3D printing technology is polycarbonate. The polymer is widely used in the polymer plastics industry, mainly due to its good mechanical properties, thermal stability, resistance over time and the aesthetics of the details obtained [12]. Moreover, in work [13], the authors presented the method of obtaining the so-called bio-PC, which has been successfully used to produce various types of toys for babies in 3D printing technology. The obtained material turned out to be environmentally friendly (56%) compared to fossil PC (0%), and its details are characterized by high strength. Unfortunately, the polymer components still do not show the expected properties; therefore, high functionality of the material can be achieved by strengthening the polymer matrix. Many scientists have attempted to combine the properties of PC with such a well-known material as acrylonitrile-butadiene-styrene copolymer [14,15,16,17,18] and poly(ethylene terephthalate) [19].

Acrylonitrile-butadiene-styrene (ABS) and polycarbonate (PC) are considered a well-known class of engineering thermoplastics due to their efficient use in 3D printing but also in automation and electronics. However, improvements in hardness, processability and thermal stability are achieved by blending ABS and PC together [15]. Kumar, M. et al. in their work [14] investigated the effect of changes in material composition on mechanical properties. The authors presented the possibility of introducing PC (from 10 to 30 wt.%) into the ABS matrix, obtaining fully compatible materials. Elsewhere [15], scientists incorporated 25–75 wt.% of the PC into the terpolymer. Each time [14,15], the mechanical strength and hardness of the composite improved along with the increase in the content of polycarbonate (PC) in the material.

However, the authors of [15] presented the possibility of obtaining composites based on poly(ethylene terephthalate)/polycarbonate (GPET/PC) copolymer intended for 3D printing. The key feature tested was the thermomechanical resistance, measured by heat deflection temperature (HDT) and Vicat softening temperature (VST), but also mechanical tests and dynamic (thermo)mechanical analysis (DMTA). Mechanical tests showed a clear trend of improving stiffness, impact strength and strength with increasing PC content in the mixes. DMTA analysis revealed significant changes in the glass transition temperature, indicating the miscibility of this type of polymer system. Moreover, scanning electron microscopy (SEM) analysis confirmed the high degree of compatibility of the GPET/PC structure.

Thermoplastics filled with carbon fiber turned out to be an interesting proposition for applications in 3D printing [20,21]. Gupta A. et al. [20] investigated the influence of printing speed, print orientation and volumetric filler content on the production process, mechanical properties (tensile strength, flexural strength, compressive strength, hardness) and surface micrography of the obtained samples. The authors presented the possibility of introducing short carbon fibers (3% to 10% by volume) into the PC matrix. The best results were obtained by a composite with a fiber content of 5.79%, a printing speed of 29.30 mm/s and a print orientation of 0° in the X-Y plane.

In summary, there are few publications in the literature on how to modify the properties of PC polymer intended for 3D printing applications; therefore, the authors of this work decided to fill this gap. As part of the work, polymer composites based on polycarbonate with the addition of selected modified nanofillers and fillers, known and described in the literature, were obtained. The introduced fillers were selected to improve the processing properties of PC, including the thermal stability and flowability, while maintaining good mechanical properties of the polymer. The influence of multiwalled carbon nanotubes on the electrical conductivity properties of composites was also investigated. In the future, the composites obtained in this way will be used for the production of selected machine elements, such as low-power gears in the technology of rapid prototyping and injection molding.

## 2. Materials and Methods

### 2.1. Materials

Commercial polycarbonate (Makrolon ET 3227, WW Ekochem Spółka z o.o. Sp.k., Głogowo, Poland) was used as a polymer matrix (designated as PC). PC was filled with: silica (S) containing aluminum oxide (Aerosil MOX 170, Evonic Industries, Hanau, Germany), bentonite (B) (“Specjal” technical product, Zębiec SA Zakłady Górniczo-Metalowe, Zębiec, Poland) modified with quaternary salt ammonium (BARQUAT^®^ DM80, Lonza, Switzerland), hybrid filler lignin (L) (Sigma-Aldrich, Burlington, MA, USA)/silicon dioxide (Syloid 244, WR Grace & Co., Baltimore, MD, USA) and multiwalled carbon nanotubes (CN) (PlasmaChem, Berlin, Germany). Detailed information on the procedure of preparing bentonite modified with quaternary ammonium salt and nanoparticles of silicon dioxide and lignin was previously patented and described [22,23] and [24,25]. Modified fillers were introduced into the PC to improve the thermal stability and flowability of the composites. Additionally, the influence of multiwalled carbon nanotubes on the electrical conductivity of PC was investigated. Hybrid fillers were introduced into the PC in order to study the synergy effect of their operation and the influence on the mechanical and rheological properties of the obtained composites. Chemically modified polyethylene grafted with maleic anhydride (Fusabond E926, DuPont, Wilmington, DE, USA) was used as a compatibilizer. The compositions of individual compositions are summarized in Table 1.

### 2.2. Preparation of the Composite

**Figure 1:**
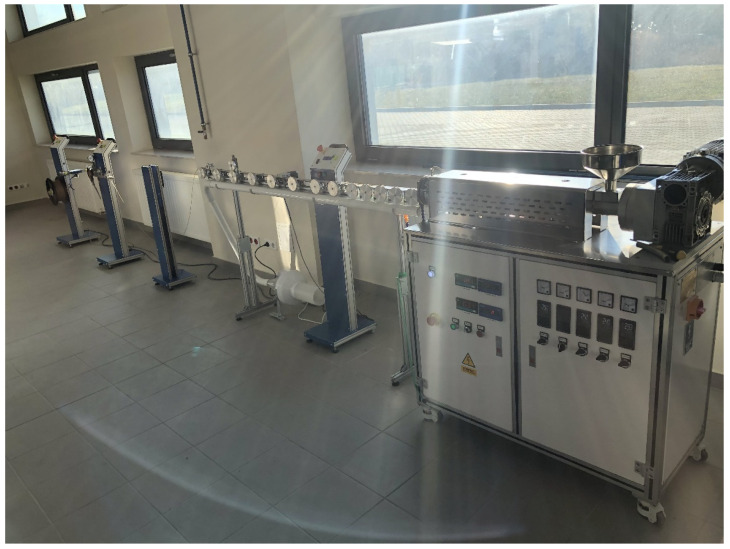
Proprietary technological line for obtaining filaments.

Before mixing the appropriate amount of the components of the individual compositions, the materials were dried under vacuum (PC: 100 °C, 4 h; fillers S, B, L: 200 °C, 24 h). The components of the composition were homogenized on a Coperion twin-screw extruder equipped with a pelletizing line using the following parameters: screw speed 400 rpm, extrusion capacity 4 kg/h, temperature: from 240 °C to 280 °C. The granules obtained in this way were dried under vacuum at 100 °C for 4 h. The dried composites were used to obtain filaments with a diameter of approx. 1.75 ± 0.05 mm using a designed line for producing filaments (manufactured by Gamart SA, Jasło, Poland) in the temperature range of extrusion from 240 °C to 270 °C (Figure 1).

### 2.3. Sample Preparation

**Figure 2:**
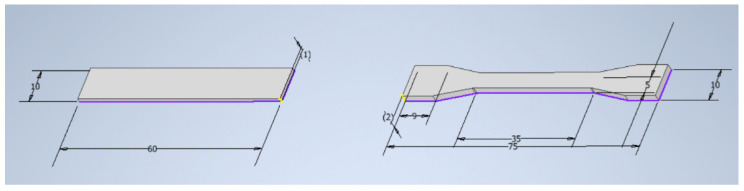
The dimensions of the samples— a bar and a paddle, respectively.

**Figure 3:**
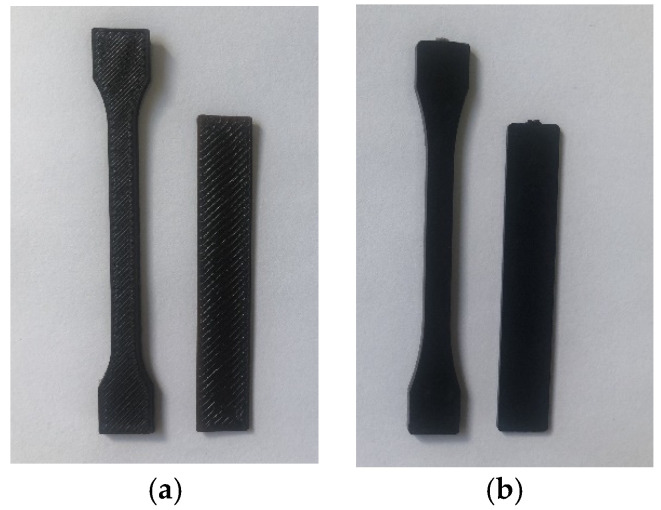
Samples obtained by the method of: (**a**) 3D printing; (**b**) injection molding.

The composites were used to obtain the samples (Figure 2 and Figure 3) needed for further tests on the UP BOX (Tiertime) 3D printer in the MEM technology and by the injection method on the Haake MiniJet II mini-injection molding machine.

The process parameters are summarized in Table 2 and Table 3.

### 2.4. Characterization

MFR, melt mass flow index, was determined using a DYNISCO 4781 Plastometer (Kayeness INC., Honey Brook, PA, USA). Samples of approximately 4 g were filled into a suitably heated apparatus, preload applied for 240 s. After that time, the load was changed to proper the proper value—2.16 kg—and measurements began. The sample extruded from the nozzle was cut every 20 s and then weighed. Three measurements were conducted for each of the series. 

Rockwell hardness was carried out using a hardness tester, Zwick/Roell (Zwick GmbH & Co., Ulm, Germany), at ambient temperature. The sample was placed in the apparatus, the specific load (load at which the indenter will sink to a thickness of 0.15–0.35 mm) was applied and the measurement began for 30 s. Ten determinations were carried out for each of the series. 

Charpy impact strength was determined using impact hammer (PSW GEHARD ZORN, Stendal, Germany) with a strength of 1 J. The test specimens were placed horizontally on the machine’s supports in such a way that the hammer hits the center of the sample edge. Five measurements were taken for each series, and the result was displayed on the camera monitor. 

The determination of strength properties during the static tensile test was performed on an INSTRON 5967 (Instron, Grove City, PA, USA) testing machine at ambient temperature. Appropriate process parameters (stretching rate, sample dimensions) were set, then the samples were placed in machine holders. The progress of the process was observed on a computer and the measurement continues until the assumed value, e.g., stress, deformation or until the sample breaks. The results were recorded in the form of chats and tables. 

Hitachi S-3400N scanning electron microscope (Red Star Vietnam Co., Ltd., Hanoi, Vietnam) was used to observe the microstructure of the materials produced. Before observation, polymer and composite samples were sprayed with a palladium gold layer. Observations were carried out using a 5 keV voltage.

A Nikon ECLIPSE LV100POL microscope equipped with a LU Plan Fluor 5x lens and camera Digital Sight DS-5Mc (Nikon Corporation, Tokyo, Japan) was used to determine the degree of porosity of samples developed with 3D printing. Prior to observation, the samples were frozen in liquid nitrogen and then cut to obtain a flat surface. From the obtained microstructure images in the MATLAB R2021a program with the addition of a porosity calculator, the degree of porosity of the polymer and composites was calculated. 

The Hommel Tester T1000 (JENOPTIK, Schwenningen, Germany) equipped with the TASTER TKL 300 L was used to determine the roughness of the samples developed by 3D printing. Scanning was carried out at a speed of 0.20 mm/s on sections with a length of 4.80 mm. 

To determine the dimensional stability of the samples (bar) obtained with 3D printing, the TOPEX caliper measurement was used (Grupa Topex Sp. z o.o. Sp.k., Warsaw, Poland). The obtained dimensions of the samples were compared to the given dimensions in the MEM process.

Thermogravimetric analysis was performed with TGA/DSC 1 (METTLER Toledo, Schwerzenbach, Switzerland) under a nitrogen atmosphere. About 5 mg of 3D-printed samples were heated on platinum dishes from 25 °C to 600 °C, at a heating rate of 10 °C/min. The results were analyzed using STAR^e^ Software (METTLER Toledo).

Differential scanning calorimetry was performed on a Mettler Toledo DSC 1 STAR^e^ System (METTLER Toledo, Schwerzenbach, Switzerland). Measurements were conducted under a nitrogen atmosphere in airtight aluminum crucibles. About 15 mg of 3D-printed samples were heated from 0 °C to 200 °C at a heating rate of 10 °C/min, then cooled to 0 °C at a heating rate of 10 °C/min, and re-heated to 200 °C at a heating rate of 10 °C/min. The results were developed in the STAR^e^ Software Default DB V16.20 program.

Wide-angle X-ray diffraction (WAXS) measurements were conducted using a NanoStar-U diffractometer (Bruker) with a two-dimensional detector in transmission geometry. X-ray radiation with a wavelength of λ 1.54 Å was produced by the radiation of a copper lamp powered by a voltage of 600 µA at 50 kV. Measurements were carried out on 3D-printed samples at room temperature (about 22 °C). The scattering angle range was from 0° to 28°.

The chemical structure was analyzed by Fourier Transform infrared spectroscopy using a Nicolet 8700 spectrophotometer (Thermo Fisher Scientific Inc., Waltham, MA, USA) with a diamond ATR attachment (Smart iTR™ Attenuated Total Reflectance). Each 3D-printed sample was scanned 128 times in the wavelength range 4000–650 cm^−1^, absorption spectra were recorded. The results were analyzed using the OMNIC Spectra software. 

Electrical conductivity was designated based on dielectric measurements conducted with broadband dielectric spectroscopy (Concept 80 System, Novocontrol GmbH, Montabaur, Germany) [26]. The investigation was performed at a constant temperature of 20 °C for samples in the form of disks 20 mm in diameter.

## 3. Results and Discussion

**Figure 4:**
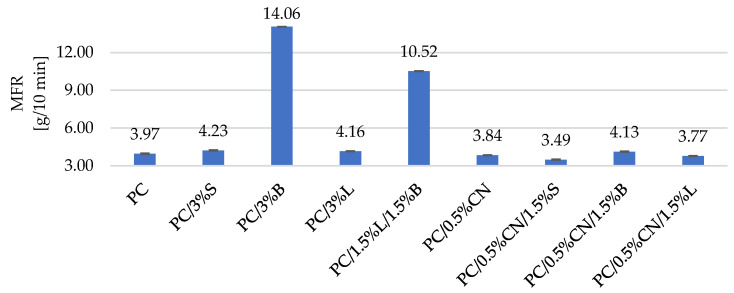
Summary of the obtained mass flow rate (MFR) results. PC—polycarbonate, S—modified silica, B—modified bentonite, L—lignin/SiO_2_ hybrid, CN—multiwalled carbon nanotubes.

The MFR index is important for the analysis of the rheological flow properties of the polymer because it significantly affects the properties of the process of obtaining specimens for functional tests [27]. The mass flow rate (MFR) also allowed determining the influence of the fillers used on the flowability of the obtained polymeric materials. On the basis of the obtained test results summarized in Figure 4, it can be concluded that composites containing modified fillers (PC/3%S, PC/3%B, PC/3%L, PC/1.5%L/1.5%B) are characterized by an increased value of the mass melt flow rate, which is consistent with the results described in the literature [28,29,30,31]. The best MFR results were obtained for the composite PC/3%B and PC/1.5%L/1.5%B, where, in comparison with the unfilled PC, MFR increased by 254.6% and 165.3%, respectively. It is known in the literature that the addition of selected modified fillers increases the mass flow rate of the tested composites (such an effect is very beneficial in the case of better filling in the injection mold and denser printing in the case of 3D printing technology) [28,29,30,31]. 

Unfortunately, we do not see this for composites containing unmodified CN filler [32,33,34]. The decrease in MFR ranges from −3.28% to −12.11% and indicates that the viscosity of the system increases after the addition of multiwalled carbon nanotubes PC/0.5%CN and the filler combination PC/0.5%CN/1.5%S, PC/0.5%CN/1.5%L [35,36]. Lowering the MFR of the tested composites containing these fillers is also described in the literature [32], but the decrease in the obtained results did not change the processing conditions and the flow behavior of PC. The reduction in MFR values can be attributed to the emerging interconnected networks of nanotubes that hinder the molecular movement of the polymer chains [27,36,37].

Analyzing the results of research on the mechanical properties of the obtained composites, it can be seen that the samples obtained by 3D printing have worse mechanical properties compared to the samples obtained by injection into the mold. The observation presented is widely described in the literature [7,38,39] and results from the greater homogeneity of the samples obtained by the injection technique [40]. 

**Figure 5:**
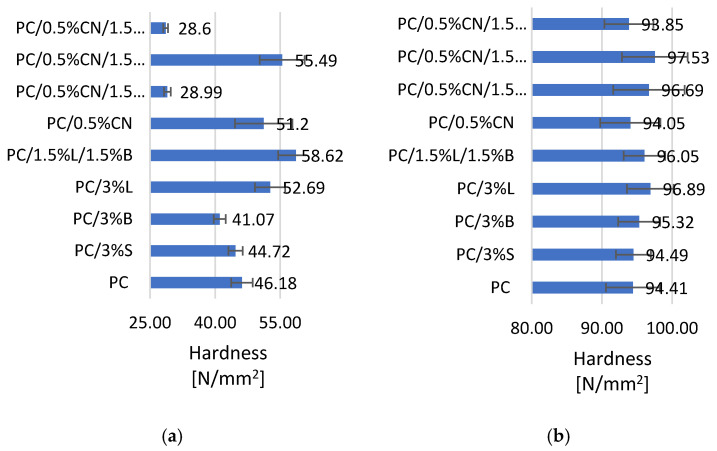
Hardness test results (**a**) samples obtained by 3D printing, (**b**) samples obtained by injection molding.

The introduction of fillers into the die increased the hardness of composites obtained by injection into the mold (Figure 5b) [41]. Only in the case of PC/0.5%CN and PC/0.5%CN/1.5%L compositions, a slight decrease in the value of the tested parameter can be observed, by 5.57% and 4.51%, respectively, compared to the unfilled polymer. Composite PC/0.5%CN/1.5%B obtained the highest result among the tested H = 97.53 N/mm^2^, while PC/3%L and PC/0.5%CN/1.5%S had a hardness of 96.89 N/mm^2^ and 96.69 N/mm^2^, respectively. However, in the case of samples obtained by 3D printing (Figure 5a) only a few of them, PC/3%L, PC/1.5%L/1.5%B, PC/0.5%CN and PC/0.5%CN/1.5%B, had a hardness better than that of PC. The highest result of 58.62 N/mm^2^ was achieved by the PC/1.5%L/1.5%B composite. The remaining composites, most likely due to the increase in surface roughness caused by the introduced fillers, especially 0.5%CN/1.5%S and 0.5%CN/1.5%L, in the MEM printing process obtained lower hardness results than PC [42]. Composites PC/0.5%CN/1.5%L and PC/0.5%CN/1.5%S are characterized by the highest surface roughness results of 18.65 µm and 15.65 µm, respectively (Table 4). The increase in roughness caused a decrease in the fluidity of the material, which is also confirmed by the discussed MFR results, which is most likely why the samples obtained from PC/0.5%CN/1.5%S and PC/0.5%CN/1.5%L composites in 3D printing obtained less precise geometry of samples (Table 4). 

**Figure 6:**
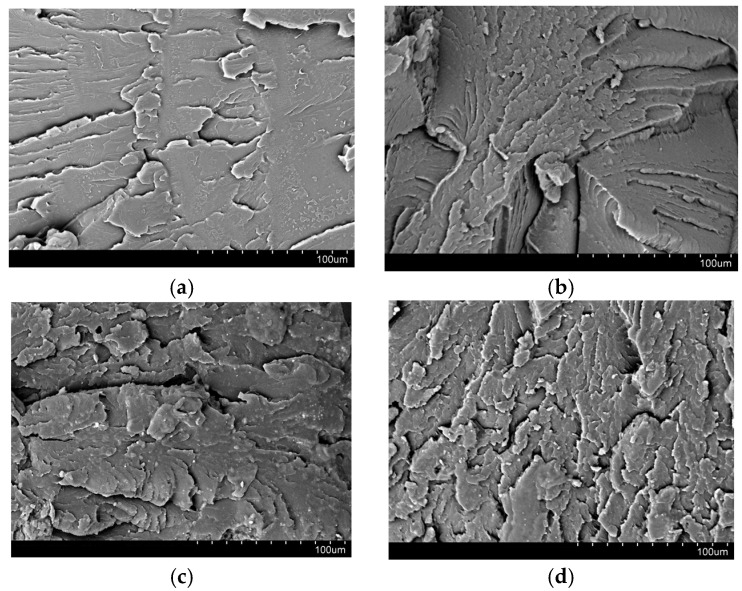

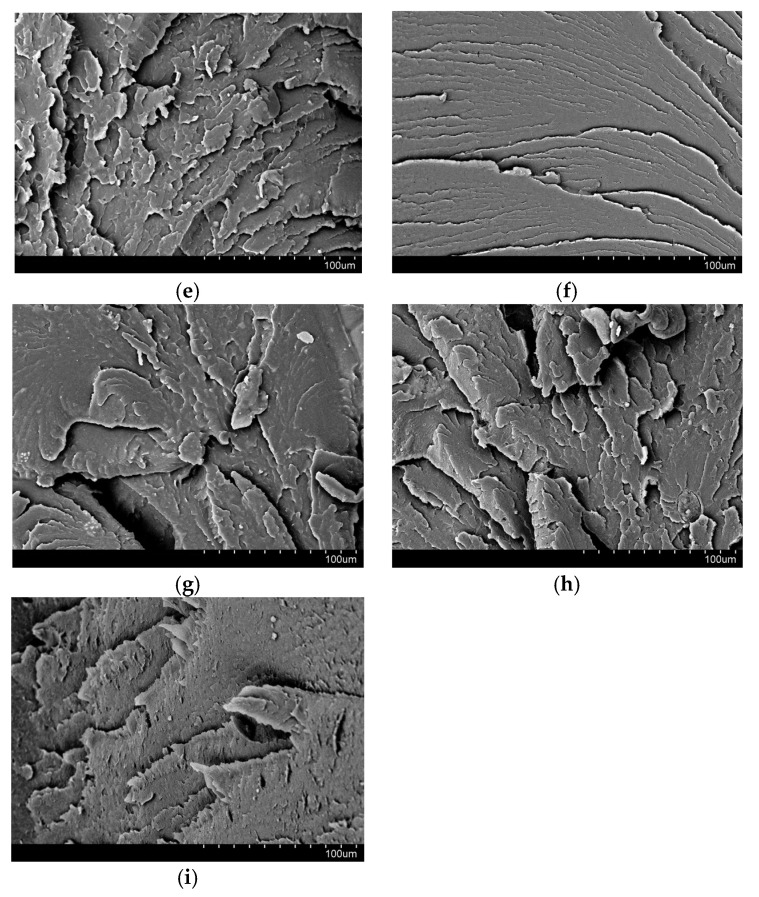
SEM test results for the composition: (**a**) PC, (**b**) PC/3%S, (**c**) PC/3%B, (**d**) PC/3%L, (**e**) PC/1.5%L/1.5%B, (**f**) PC/0.5%CN, (**g**) PC/0.5%CN/1.5%S, (**h**) PC/0.5%CN/1.5%B, (**i**) PC/0.5%CN/1.5%L.

**Figure 7:**
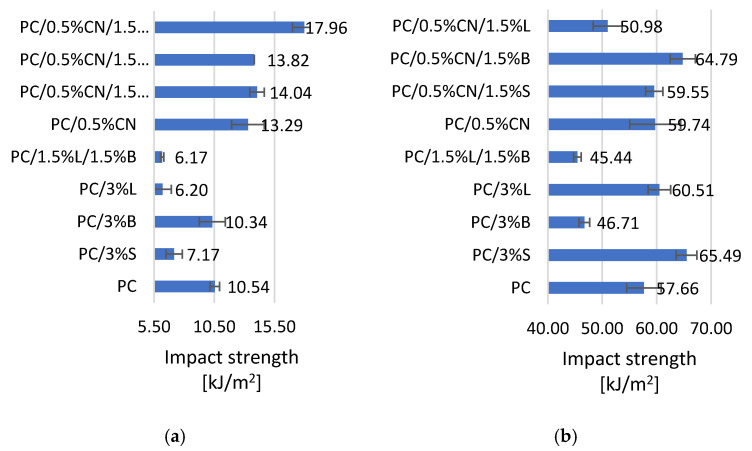
Impact test results of (**a**) samples obtained by 3D printing, (**b**) samples obtained by injection molding.

Based on the results of the impact tests, it was found that both the content and the type of filler had an effect on the tested property. For both the shapes obtained in the 3D printing technology and injection molding, the introduction of multiwalled carbon nanotubes into the PC matrix increased the material’s resistance to cracking under dynamic impact. Multiwalled carbon nanotubes have a large specific surface, when properly dispersed in the polymer matrix, they have better load-carrying capacity and better adhesive properties, which probably improves the impact toughness results, such a phenomenon has also been described in the literature [41]. The use of an optimized method of dispersing the fillers in the thermoplastic polymer matrix allowed avoiding the formation of agglomerates in the polymer matrix (which we do not observe in the SEM photos (Figure 6)), and as it is known from the literature, the presence of filler agglomerates significantly deteriorates the impact strength of composites [43]. Such an effect of proper dispersion of fillers was obtained thanks to the use of an appropriate configuration of plasticizing systems in a twin-screw extruder. The impact strength of composites with the addition of nanotubes improved by 12.7% for injection molded samples, and up to 70.4% for 3D-printed samples (Figure 7a,b). The addition of the remaining selected fillers to PC: 3%S, 3%B, 3%L and 1.5%L/1.5%B resulted in a reduction in the fracture toughness of composites compared to unfilled polymer. It may be related to the porosity of the structure, because the introduction of 3%L and 3%S resulted in obtaining 3D-printed samples with higher porosity than PC, 0.24% and 0.14%, respectively (Table 4). The exceptions are composites obtained by the injection method PC/3%S and PC/3%L, which had better impact resistance (Figure 7b). It should be mentioned that analogous results of impact tests for composites containing selected fillers are described in the literature [43,44]. 

**Figure 8:**
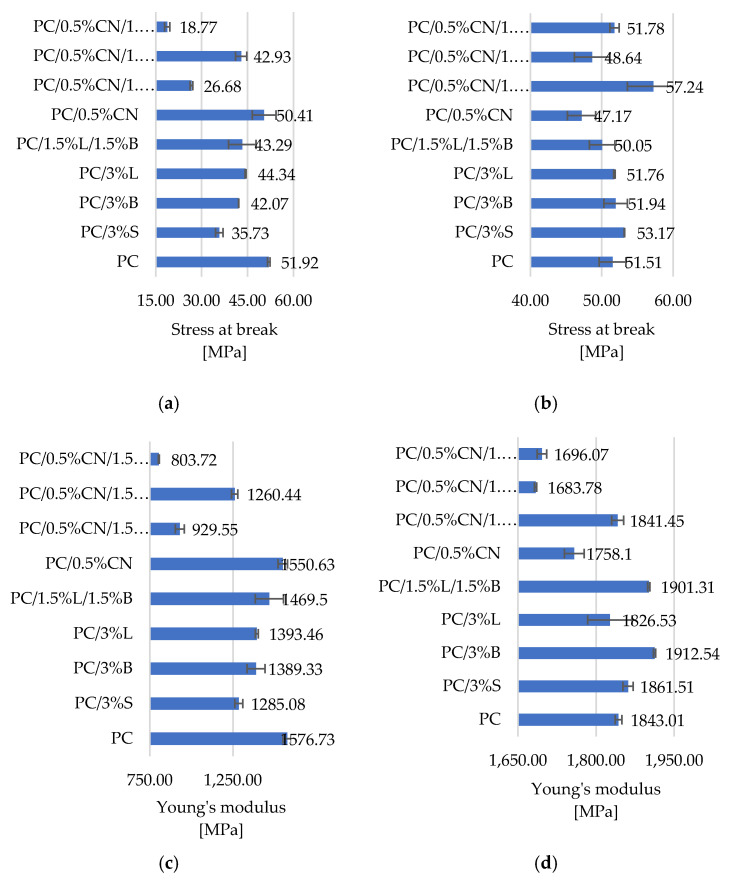

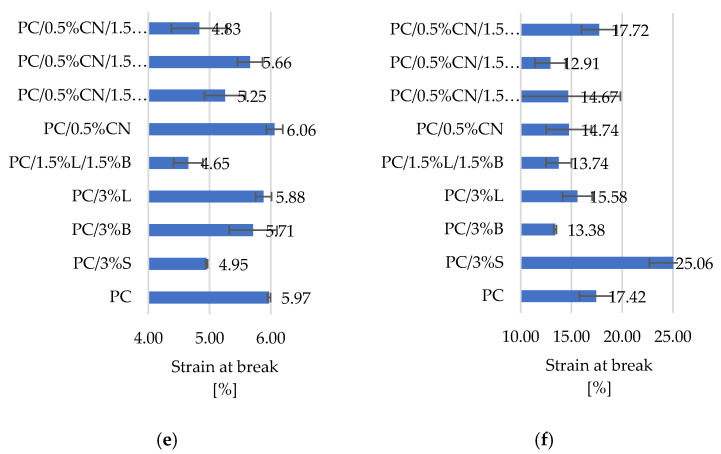
Results of static tensile strength tests: stress at break (**a**) samples obtained by 3D printing, (**b**) samples obtained by injection molding, Young’s modulus (**c**) samples obtained by 3D printing, (**d**) samples obtained by injection molding, strain at break (**e**) samples obtained by 3D printing, (**f**) samples obtained by injection molding.

Analyzing the results of the static tensile strength tests listed in Figure 8, it was observed that the addition of CN causes a decrease in the Young’s modulus of the samples, regardless of the technique of their production, which correlates with the results of hardness and impact strength and confirms the reduction in the stiffness of these materials. We also observe a decrease in stress at break for these tested composites [45]. The obtained results for the shapes obtained by the 3D method may be directly related to the increase in the degree of porosity after the introduction of CN into the polymer, but also to the lower dimensional stability of composites containing CN compared to PC (Table 4). In contrast, the injection molded composites containing the remaining modified fillers showed an increase in Young’s modulus compared to unfilled PC (1576.73 MPa). At the same time, it was observed that the introduction of these additives into the polymer matrix resulted in an increase in the flexibility of the composites obtained with the MEM technique (the obtained moldings are characterized by high dimensional stability and low porosity, except for the PC/3%L composite). In the course of testing the resistance to static tensile, so-called brittle fracture was observed for this group of composites, which is also confirmed by the results of SEM tests (Figure 6) and literature reports [42]. This phenomenon is reflected in the determination of the strain at break which has decreased compared to PC. The exceptions are PC/0.5%CN composites obtained by 3D printing and PC/3%S and PC/0.5%CN/1.5%L by injection method, for which the deformation increased by 1.51%, 43.86% and 1.72%, respectively.

The morphology of brittle fractures of the obtained composites was analyzed using scanning electron microscopy (SEM). The fractures were obtained after cooling the samples in dry ice and their impact fracture, and the results of these observations are presented in Figure 6. Based on SEM micrographs of brittle fractures of the tested samples, it was found that in the fracture of unfilled PC (Figure 6a) furrows. The addition of modified silica fillers (3%S), bentonite (3%B), lignin (3%L) and a hybrid lignin/bentonite filler (1.5%L/1.5%B) makes the breakthrough of the samples character of larger and smaller ragged plates and it is difficult to distinguish phases (polymer and filler). This morphology is most likely due to the nature of the filler used. In the case of the modified silica (Figure 6b), no agglomerates of this filler were observed in the polymer matrix, which proves a well-chosen homogenization method [46]. On the other hand, as it is known from the literature [47,48], the morphology presented in Figure 6c results from the layered structure of the modified bentonite and its organophilic nature, which facilitates good miscibility with the polymer matrix. The addition of lignin modified with silica to the polymer matrix resulted in the formation of finer, jagged plates with small protrusions [22,25]. The introduction of a hybrid system of lignin/bentonite fillers into the matrix did not cause any significant changes. We observe a reduction in finer tiles with protrusions, and instead, the formation of larger tiles, probably due to the presence of bentonite. To summarize, the fine-plate structure of these composites probably proves that we obtained nanocomposites. On the basis of successive SEM micrographs, brittle fractures were found in the fracture of PC with the addition of CN (Figure 6f), similarly to unfilled PC (Figure 6a), also a brittle fracture with small furrows. The addition of a modified silica to such a composite resulted in the creation of a larger number of large plates covered with nanoparticles probably derived from the modified silica, which was observed in the case of the PC/3%S composite. On the basis of successive SEM photomicrographs of brittle fractures of the composite with the addition of bentonite and modified lignin (Figure 6h,i), as in the case of PC/3%B and PC/3%L composites, we observe an increase in the number of plaques, and in the case of modified lignin, the edges have tiny, jagged tabs. 

**Figure 9:**
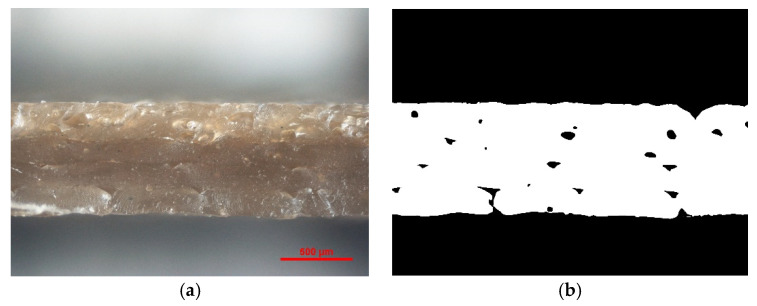
PC/3%B microstructure: (**a**) microscope image, (**b**) image from porosity calculation program.

The cross-section of the samples was examined to determine the degree of porosity of the polymer and composites (Figure 9). The obtained results are summarized in the Table 4. The introduced fillers to the PC resulted in significant changes in the test results. Composites containing modified bentonite PC/3%B, PC/1.5%L/1.5%B and PC/0.5%CN/1.5%B are characterized by the lowest porosity. Most likely, it is directly related to the flowability of the materials, as the addition of B caused a significant increase in the MFR of the composites. More fluid materials also allow obtaining samples with fewer voids between filament threads.

The introduction of the remaining fillers or filler systems to PC resulted in an increase in the porosity value of composites. The highest result was obtained for PC/3%L, which is 0.24%.

The surface quality of samples obtained in 3D technology was analyzed by means of a roughness test. When analyzing the obtained results of the Ra parameter (Table 4), it can be noticed that the introduced fillers affect the surface quality of the samples. The introduction of modified fillers 3%S, 3%B and 3%L increased the surface roughness of the polymer by 383.13%, 353.01% and 381.33%, respectively. On the other hand, the introduced hybrid filler 1.5%L/1.5%B resulted in a smoothing of the PC surface, because the PC/1.5%L/1.5%B composite has a roughness of 1.29 µm. The addition of multiwalled carbon nanotubes also allowed obtaining a lower roughness compared to PC. However, the introduced hybrid systems containing CN significantly increased the surface roughness of the composites, the highest result of 18.65 µm was obtained for PC/0.5%CN/1.5%L.

It should be noted that before developing the samples described in this article, the parameters of the printing process were optimized, including, among others, the printing angle, layer thickness, printing speed, fill factor and printing temperature, along with the temperature of the working table. The quality of the dimensions of the samples was determined by measuring the 3D-printed bars. The obtained results are a consequence of arranging the samples on the worktable during the MEM process. The samples were printed flat with dimensions of 60.00 mm and 10.00 mm on the print bed of the printer. Usually, in 3D printing technology, the first few print layers are wider than the given dimensions, because we select the temperature of the working table to ensure the adhesion of the samples. During the printing process, the elements cannot detach from the printer’s working table. This flattens the material to a thicker line. The phenomenon can be observed in Figure 9. Therefore, the dimension of the given thickness of 1.00 mm is lower in each case, and the other dimensions are correspondingly higher. By analyzing the results obtained in the Table 4, it can be concluded that the samples with the highest difference between the given and the obtained dimensions are also characterized by the highest roughness: PC/0.5%CN/1.5%L, PC/0.5%CN/1.5% S and PC/0.5%CN/1.5%B. On the other hand, the composite with the lowest roughness, PC/1.5%L/1.5%B, has the highest dimensional stability.

**Figure 10:**
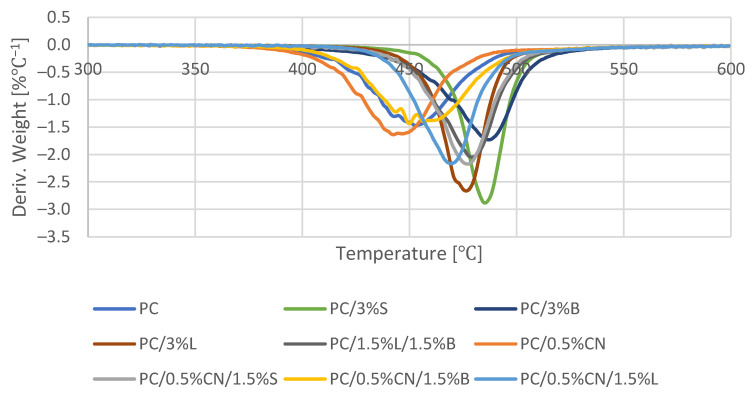
Thermograms of DTG.

The results of the tests of the thermostability properties of the mixtures are summarized in Table 5. The 5% mass loss temperature (T_5%_) was determined from the TGA curve, which can be assumed as the beginning of the degradation process. The maximum temperature of the subsequent degradation stages (Tmax)_i_ and the loss of the initial mass of the sample at the maximum temperature (Δm)_i_ were determined from the mass change derivative curve (Figure 10). 

The polymer and all composites are characterized by a single-stage thermal decomposition. The degradation process step (Figure 10) shows a slight intense peak. This peak appears in the temperature range of 375–525 °C, during which there is a loss in the range from 38% for PC/0.5%CN to 48% for PC/3%B of the initial sample mass (Δm_1_) for the tested composites (Table 5). The unmodified polymer is characterized by high thermal stability as the PC decomposition begins at 410 ℃. The introduced additives increased the thermal stability of the material for composites, except for PC/0.5%CN. This indicates a positive interfacial interaction between the filler and the PC matrix [49]. Such a phenomenon proves the appropriate dispersion of additives in the polymer matrix, which is also confirmed by the analysis of mechanical properties and SEM. The increase in the thermal stability value reflects the low thermal conductivity of the S, B and L additives. The highest result was obtained for the PC/3%S 453.17 ℃ composite, which may be related to the relatively large surface of the silica filler. The presence of 3%S in the polymer matrices leads to the creation of an interlayer zone on the filler surface, and thus to the immobilization of polymer chains on the additive surface [24,25]. Moreover, the presence of 3%L also substantially increases thermal stability, the beginning of the 445 ℃ degradation process, which may be related to the formation of aromatic charred lignin radicals that form a protective coating, and therefore, the material decomposes at higher temperatures [25]. Similarly selected hybrid fillers 0.5%CN/1.5%S and 0.5%CN/1.5%L increased thermal stability. The introduced multiwalled carbon nanotubes were not functionalized or modified prior to mixing, and therefore the TGA curves for the PC and PC/0.5%CN samples showed only a slight difference. The decrease in the value of thermal stability is a reflection of the high thermal conductivity of nanotubes, which can form locally high temperatures in their larger CN clusters, which was not observed in the case of the PC polymer matrix [50]. Observing the CN-containing composite (PC/0.5%CN), it showed greater weight loss (Table 5) due to the low overheating effect [51].

**Figure 11:**
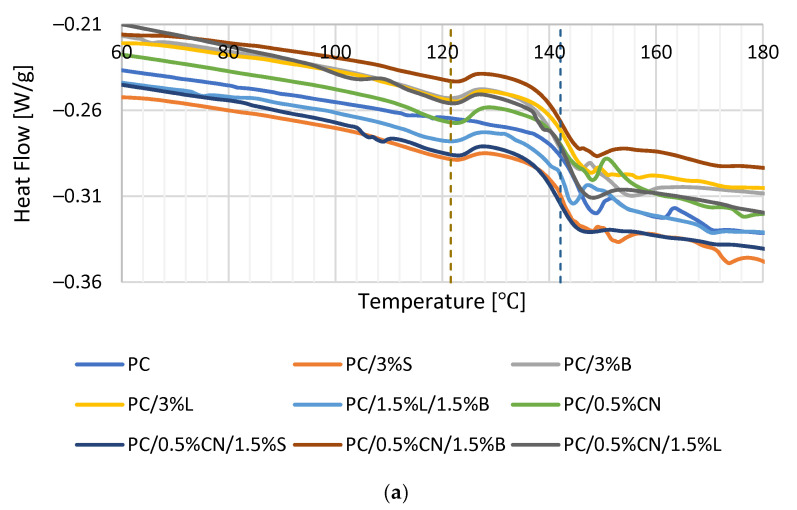

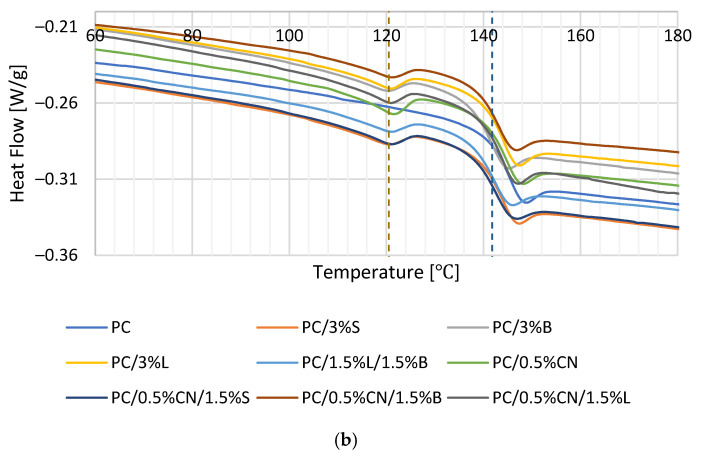
First heating (**a**) and second heating (**b**) curves for the composites.

The thermal effect of the materials was investigated using a DSC calorimeter. The thermograms are characterized by the appearance of an endothermic peak at about 144 °C, which can be attributed to the glass transition temperature (Tg) of the PC phase (Figure 11). The obtained result is confirmed by data obtained by other scientists [49,52,53]. Maintaining the same Tg values for all tested systems during the first and second heating (Table 6) indicates that neither the content nor the modification of the filler surface affects the mixability [50]. Analyzing the thermograms obtained for the composites, one can also observe the second endothermic peak at a temperature of about 122 °C, which corresponds to the melting point of the added additive. For all composites containing different fillers, a transition at this temperature was observed, and therefore, the peak most likely comes from the compatibilizer introduced into the polymer matrix, polyethylene grafted with maleic anhydride, more so as polyethylene has a melting point of about 120° [54,55].

**Figure 12:**
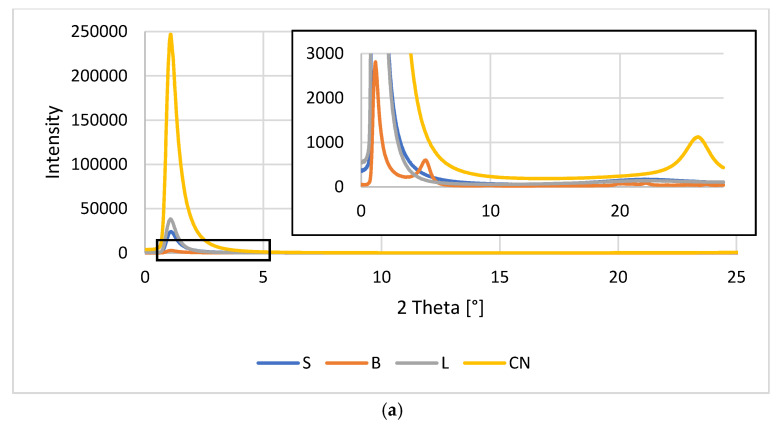

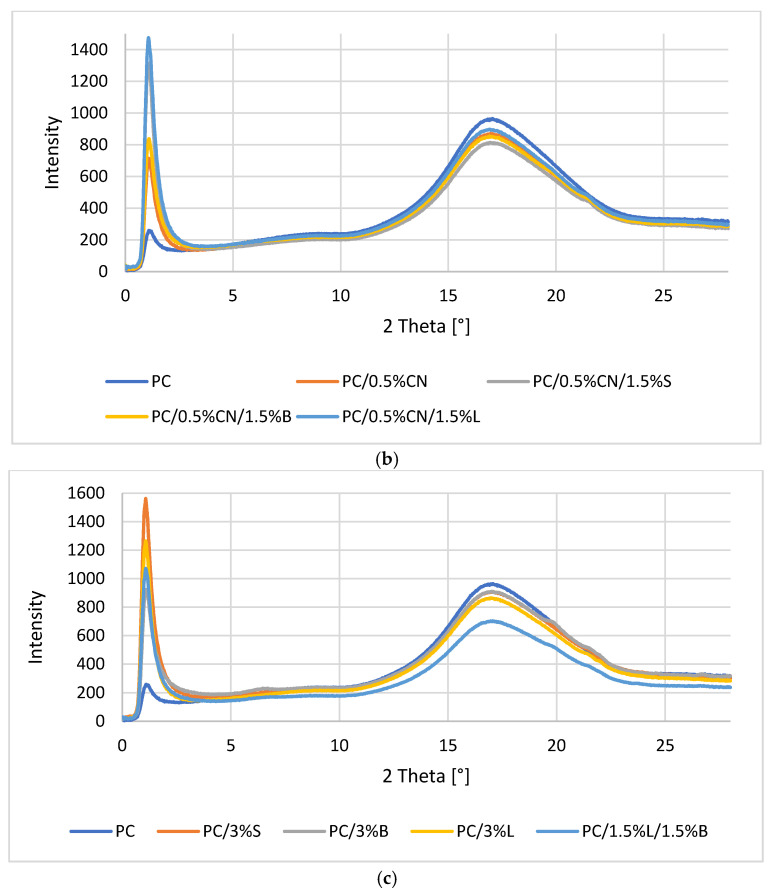
WAXS patterns: (**a**) fillers; (**b**) PC and composites with the addition of modified S, B, L and CN fillers; (**c**) PC and composites with the addition of modified fillers S, B and L.

**Figure 13:**
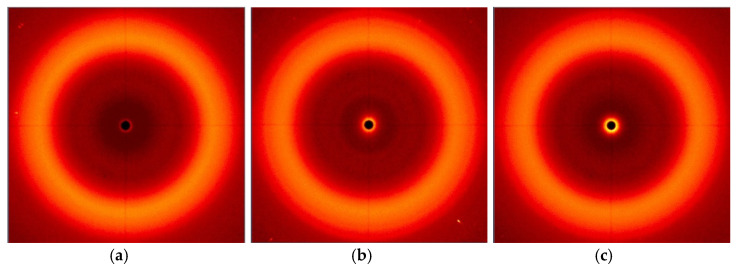

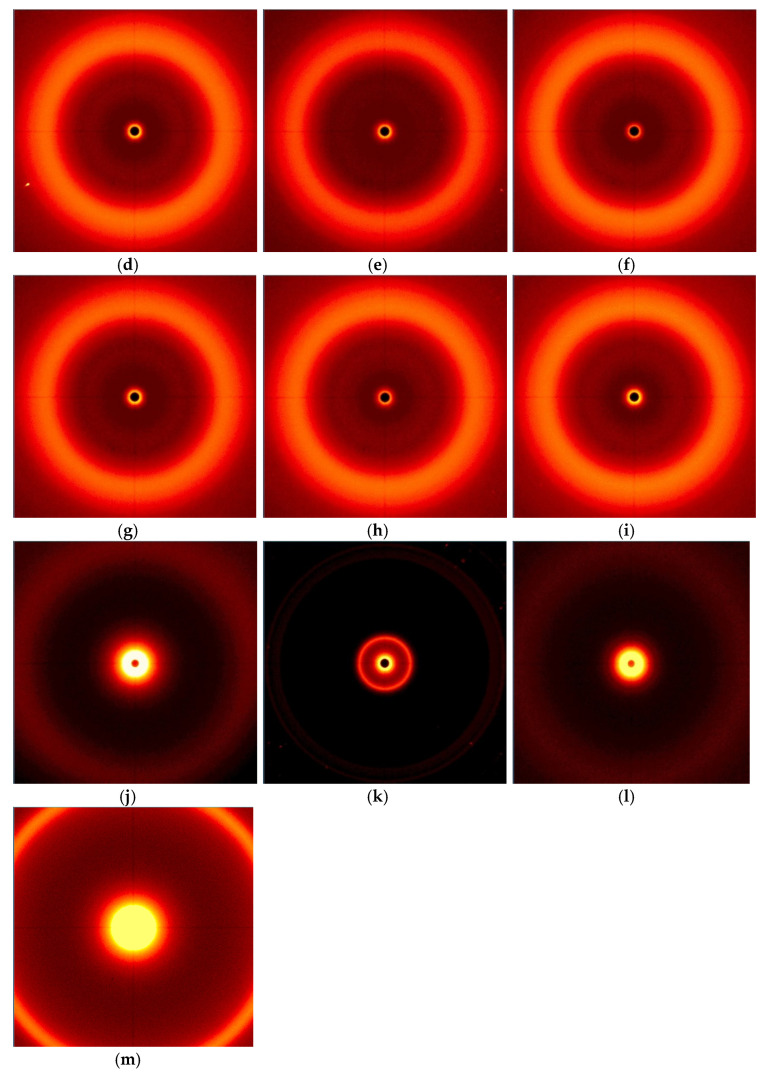
Two-dimensional WAXS images: (**a**) PC; (**b**) PC/3%S; (**c**) PC/3%B; (**d**) PC/3%L; (**e**) PC/1.5%L/1.5%B; (**f**) PC/0.5%CN; (**g**) PC/0.5%CN/1.5%S; (**h**) PC/0.5%CN/1.5%B; (**i**) PC/0.5%CN/1.5%L; (**j**) S; (**k**) B; (**l**) L; (**m**) CN.

The morphology and molecular orientation of PC, composites and fillers were characterized by WAXS analysis. Plots of radiation intensity as a function of scattering angle are shown in Figure 12, and wide-angle 2D X-ray diffraction patterns are shown in Figure 13. 

The scattering angle range for the tested materials was from 0° to 28°. In this range, the radiation intensity as a function of the scattering angle was observed only for bentonite B fillers and CN multiwalled carbon nanotubes (Figure 12a). The 2D WAXS images (Figure 13j,l) also show the presence of S and L additions in this range, however, the obtained intensity was too low to record their scattering angle. The remaining graphs show the results of the obtained composites, respectively, containing modified fillers and multiwalled carbon nanotubes (Figure 12b) and only modified fillers (Figure 12c).

The distance between successive planes of the filler (d_hkl_) was calculated from the Bragg formula:

(1) $$d_{\mathrm{hkl}}=\frac{n\lambda }{2sin\theta },$$

where n is the degree of diffraction (*n* = 1, 2...), λ is the wavelength of radiation used and 2θ is the angle at which the diffractive peak occurs, as read from the WAXS graph.

The particle size in the Scherrer formula was also determined:

$$D_{\mathrm{hkl}}=\frac{K\lambda }{bcos\theta }.$$

where D_hkl_ is the reflex width dependent on the size of crystallites, K is Scherrer’s permanent, K = 1, λ is the wavelength of radiation used and b is the half-width of the diffraction peak for the plane (_hkl_).

Analyzing the results obtained for the fillers (Figure 12a), we can see a peak for B at 4.98°, which can be attributed to the bentonite sheet spacing (001) [47,48], while the peak for CN is at 26.02°, attributable to the spacing of the graphite sheets (002) [56]. The introduced fillers are well dispersed in the polymer matrix, because the graphs derived from the obtained composites are characterized by one wide peak with a value of 2θ amounting to about 17°, which is a characteristic result for the arrangement of the polymer PC 2θ = 17.1° [57]. The distances between successive packets of filler plates and the size of their particles were calculated. F for B, d_khl_ is 18.20 Å and D_khl_ is 110.8 Å, while CN is characterized by a d_khl_ value of 3.51 Å and D_khl_ equal to 55.8 Å. It should be noted that the lack of filler peaks in the WAXS data for composites is also attributed to good dispersion of additives in the PC matrix [58].

**Figure 14:**
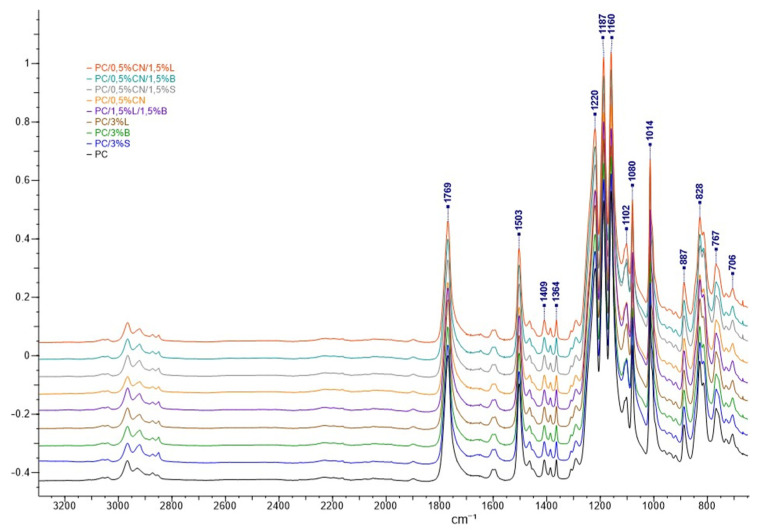
FT-IR spectra recorded for the composition.

Fourier Transform infrared spectroscopy allows characterizing chemical bonds and analyzing the frequency of compounds at the atomic level [59]. Figure 14 shows similar absorbance peaks containing all the characteristic PC bands for the polymer and its composites. The spectra contain molecular vibrations typical for PC, at 2980 cm^−1^, 2920 cm^−1^ and 887 cm^−1^, while 828 cm^−1^ and 767 cm^−1^ can be attributed to the C-H stretching vibrations of the -CH_3_ groups, and 1769 cm^−1^ and 1014 cm^−1^ are derived from molecular vibrations -C=O [59,60,61]. The peaks at 1503 cm^−1^ can be attributed to molecular vibrations of the aromatic ring, at 1409 cm^−1^ and 1364 cm^−1^ to deformation -OH, and 1102 cm^−1^, 1160 cm^−1^, 1180 cm^−1^, 1187 cm^−1^ and 1220 cm^−1^ are attached to the acetyl group -C-O [61,62]. There are no new bands on the spectrum that could be derived from chemical bonds of the added additives, regardless of the type of filler; the spectra do not differ much, most probably due to too low amounts of fillers (maximum 3 wt.%).

**Figure 15:**
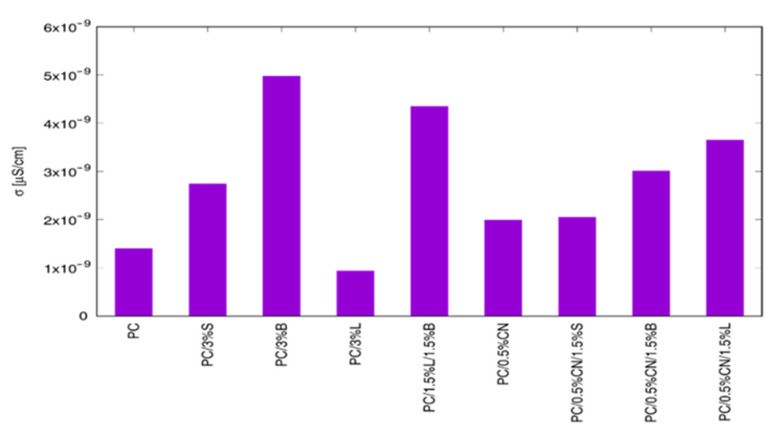
Electrical conductivity of the obtained composites.

The electrical properties of PC-based nanocomposites with various types of fillers were studied with broadband dielectric spectroscopy. On the basis of AC conductivity spectra, as its value at the lowest tested frequency (0.1 Hz), the DC electrical conductivity was determined [26,63]. Obtained results were presented in Figure 15 where a favorable filler effect on electrical conductivity is visible for all measured samples. The only exception is PC/3%L nanocomposite where a slight decrease is noted. The highest increase shows PC/3%B where enhancement was over 350%; additionally, PC/1.5%L/1.5%B nanocomposite is characterized by an increase in electrical conductivity higher than 300%. On the other hand, nanocomposites with carbon nanotubes show relatively low enhancement, not exceeding 143% for PC/0.5% or 260% for PC/0.5%CN/1.5%L.

## 4. Conclusions

Research was conducted on the development of polymer composites with a PC matrix with the addition of modified nanofillers for MEM technology. For this purpose, several fillers known and described in the literature were selected, which were dispersed in the PC matrix, and then a material for 3D printing—filaments were obtained from the composites developed in this way—with the use of the designed technological line. The influence of modified fillers, including silica modified with alumina, bentonite modified with quaternary ammonium salt and hybrid lignin/silicon dioxide filler, but also unmodified multiwalled carbon nanotubes on the properties of the obtained polycarbonate (PC) composites, was investigated. It was found that the addition of modified fillers to the polymer matrix increased the flowability of the material (MFR). The highest result was obtained for composite PC/3%B, where the change compared to unfilled PC is 254.6%. On the other hand, the addition of multiwalled carbon nanotubes resulted in a reduction in the flow index from 3.28% to 12.11%. An increase in the hardness according to Rockwell of the obtained composites was observed, both for injection molded and 3D-printed moldings; only MEM moldings with PC/0.5%CN/1.5%S and PC/0.5%CN/1,5%L obtained a lower hardness of 28.99 N/mm^2^ and 28.6 N/mm^2^, respectively. The addition of multiwalled carbon nanotubes increased the impact strength according to Charpy. Unfortunately, composites containing only modified fillers show a decrease in the obtained impact toughness values. It was also observed that the stiffness of the material decreased with the addition of multiwalled carbon nanotubes as indicated by a decrease in Young’s modulus and a corresponding increase in tensile stress for moldings obtained by injection molding. On the other hand, the remaining composites showed an increase in stiffness compared to PC (1576.73 MPa). Observations of the microstructure of composites using the SEM method confirmed the uniform distribution of fillers in the polymer matrix, which was also observed on the basis of the results of the WAXS analysis. The porosity results show that the introduced fillers affect the structure of the samples obtained in 3D. The PC/3% B composite has the most homogeneous structure. The introduced fillers also affected the surface roughness results of the samples obtained with 3D printing, the lowest roughness of 1.29 µm is characterized by PC/1.5%L/1.5%B. Satisfactory dimensional stability of the samples obtained in the MEM technology was obtained. The TGA results show that the addition of fillers increased the thermal stability of the composites; only in the case of PC/0.5%CN and PC/0.5%CN/1.5%B was the temperature of the onset of degradation lower compared to PC. DSC revealed that PC is characterized by typical phase changes for the material, and the introduced additives did not change the thermal history of the composites. The spectrum obtained for the material (FT-IR) contains all the characteristic functional groups of the material, and the introduced fillers did not affect the distribution of the bands obtained. The electrical conductivity of PC-based composite was investigated and the impact of various filler was presented. The highest enhancement in electrical conductivity was observed for PC/3%B and it was over 350%.

## Figures and Tables

**Table 1:** Compositional data of the composites.

Composition	PC Content (wt.%)	Silica Content (wt.%)	Lignin/SiO_2_ Content (wt.%)	Bentonite Content (wt.%)	Fusabond Content (wt.%)	Multiwalled Carbon Nanotubes Content (wt.%)
PC	100	-	-	-	-	-
PC/3%S	96	3	-	-	1	-
PC/3%B	96	-	-	3	1	-
PC/3%L	96	-	3	-	1	-
PC/1.5%L/1.5%B	96	-	1.5	1.5	1	-
PC/0.5%CN	98.5	-	-	-	1	0.5
PC/0.5%CN/1.5%S	97	1.5	-	-	1	0.5
PC/0.5%CN/1.5%B	97	-	-	1.5	1	0.5
PC/0.5%CN/1.5%L	97	-	1.5	-	1	0.5

**Table 2:** Selected printing parameters.

Nozzle diameter	0.4 mm
Nozzle material	Brass/TwinClad XT
Layer height	0.2 mm
Infill percentage	100%
Infill pattern	Rectilinear ± 45
Extrusion temperature	270 °C
Bed temperature	80 °C
Bed surface	Up Flex
Printing speeds	70 mm/s

**Table 3:** Selected injection parameters.

	Paddles	Bars
Mold temperature, °C	90	90
Injection temperature, °C	280	280
Injection pressure, bar	650	950
Post pressure, bar	600	900
Plasticizing time, s	180	180
Injection time, s	5	5
Post time, s	3	3

**Table 4:** Summary of the results of porosity, roughness and dimensional stability tests of samples obtained by 3D printing.

Samples	Porosity (%)	Ra (µm)	Dimensional Stability (60.00 mm × 10.00 mm × 1.0 mm)
PC	0.10	1.66 ± 0.08	**60.09** ± 0.13 × **10.09** ± 0.12 × **0.92** ± 0.81
PC/3%S	0.14	8.02 ± 0.70	**60.05** ± 0.01 × **10.08** ± 0.01 × **0.95** ± 0.01
PC/3%B	0.05	7.52 ± 0.04	**60.12** ± 0.01 × **10.08** ± 0.01 × **0.88** ± 0.06
PC/3%L	0.24	7.99 ± 1.30	**60.14** ± 0.06 × **10.06** ± 0.01 × **0.95** ± 0.01
PC/1.5%L/1.5%B	0.07	1.29 ± 0.08	**60.06** ± 0.13 × **10.06** ± 0.11 × **0.96** ± 0.01
PC/0.5%CN	0.17	1.51 ± 0.04	**60.04** ± 0.01 × **10.09** ± 0.01 × **0.87** ± 0.01
PC/0.5%CN/1.5%S	0.13	15.65 ± 1.68	**60.08** ± 0.01 × **10.08** ± 0.04 × **0.81** ± 0.01
PC/0.5%CN/1.5%B	0.03	9.12 ± 1.06	**60.05** ± 0.07 × **10.09** ± 0.01 × **0.86** ± 0.02
PC/0.5%CN/1.5%L	0.13	18.65 ± 0.85	**60.10** ± 0.02 × **10.09** ± 0.03 × **0.82** ± 0.04

**Table 5:** The results of research on the properties of thermostability of composites.

	Parameter	T_5%_, °C	Tmax_1_, °C	Δm_1_, _%_	R_600_, _%_
Filament					
PC		410.00	453.17	40.32	21.83
PC/3%S		**453.17**	485.83	43.11	22.20
PC/3%B		422.83	**487.00**	**48.26**	21.86
PC/3%L		445.00	476.50	43.91	20.88
PC/1.5%L/1.5%B		438.00	478.83	43.95	21.43
PC/0.5%CN		**403.00**	**442.67**	**37.94**	**17.05**
PC/0.5%CN/1.5%S		442.67	477.67	44.95	20.51
PC/0.5%CN/1.5%B		407.67	456.67	42.32	17.19
PC/0.5%CN/1.5%L		440.33	469.50	41.39	**22.21**

**Table 6:** Summary of *T*g and Delta *C*p changes after the first and second heating steps of the individual compositions.

Composition	*T*g (°C) (First Heating)	Delta *C*p (J/g·K)	*T*g (°C) (Second Heating)	Delta *C*p (J/g·K)
PC	**143.40**	0.17	143.25	0.24
PC/3%S	142.03	0.19	142.70	**0.25**
PC/3%B	141.03	0.26	**140.47**	0.22
PC/3%L	142.77	0.25	142.38	0.22
PC/1.5%L/1.5%B	142.58	**0.13**	141.25	**0.19**
PC/0.5%CN	142.20	0.17	**143.48**	0.22
PC/0.5%CN/1.5%S	**140.92**	0.24	140.99	0.21
PC/0.5%CN/1.5%B	141.40	0.21	141.42	0.10
PC/0.5%CN/1.5%L	142.72	**0.27**	142.25	0.22

## Data Availability

The data presented in this study are available on request from the corresponding author.
